# Synthesis of Some Monoazo Disperse Dyes Derived from Aminothienochromene

**DOI:** 10.3390/molecules18088837

**Published:** 2013-07-25

**Authors:** Saleh Mohammed Al-Mousawi, Morsy Ahmed El-Apasery

**Affiliations:** 1 Chemistry Department, Faculty of Science, Kuwait University, P.O. Box 5969, Safat 13060, Kuwait; 2 Dyeing, Printing and Textile Auxiliaries Department, Textile Research Division, National Research Centre, Dokki, Giza 12622, Egypt; E-Mail: elapaserym@yahoo.com

**Keywords:** polyester fabrics, disperse dyes, aminothienochromene, MOREtechnology

## Abstract

A series of azo disperse dyes based on aminothienochromene were synthesized. The fastness properties of the dyed samples were measured. Most of the dyed fabrics tested displayed excellent washing and perspiration fastness and moderate light fastness.

## 1. Introduction

The chemistry of aminothiophenes has received much attention due to their facile availability through the versatile Gewald synthesis [1]. The importance of azothiophene dyes is demonstrated by the high number of publications and patents [2,3] dealing in one way or another with that class of dyestuffs, noted for their highly interesting dyeing properties, namely their high degree of brightness compared with azo dyes derived from anilines [4] and their excellent shade brightness [5]. Their derivatives have long been used as intermediates in the dyestuff industry [6,7,8,9].

Aminothienochromene compounds are very useful as precursors for the synthesis of fused heterocyclic ring systems, which display an important range of biological and pharmacological activities [10,11,12,13,14]. In spite of the large number of reports on the utility of these compounds in the dye industry, to our knowledge, their corresponding arylazothienochromenes have never been reported as potential monoazo disperse dyes.

In view of these findings, and in continuation of our previous studies [14] on the synthesis of a variety of aminothienochromene dyes, we now report on the successful synthesis of arylazothienochromene dyes from readily obtainable cheap starting materials and their applications as disperse dyes for dyeing polyester fabrics.

## 2. Results and Discussion

In conjunction with our interest in developing efficient routes to polyfunctional heteroaromatics as disperse dyes for polyester fabrics and/or Dye Diffusion Thermal Transfer (D2T2) printing, we report here our results on the reactivity of aminothienochromene toward some aryldiazonium chlorides. As we have placed emphasis in the last few years on adopting microwave heating as a suitable substitute to conventional heating in water or an oil baths [14], we have utilized heating in a direct beam microwave oven. We observed that by adopting Microwave Organic Reactions Enhancement (MORE) technology in the reaction of *o*-hydroxyacetophenone with ethyl cyanoacetate and elemental sulfur in the presence of diethylamine as basic catalyst with a focused microwave heating oven as an energy source at 50 °C for 4 h, [15], 3-aminothienochromene (**4**) is produced in 48% yield. It is noteworthy that compound **4** was prepared in the literature in 37% yield by heating for 24 h utilizing morpholine or diethylamine as a basic catalyst [16].

**Scheme 1:**
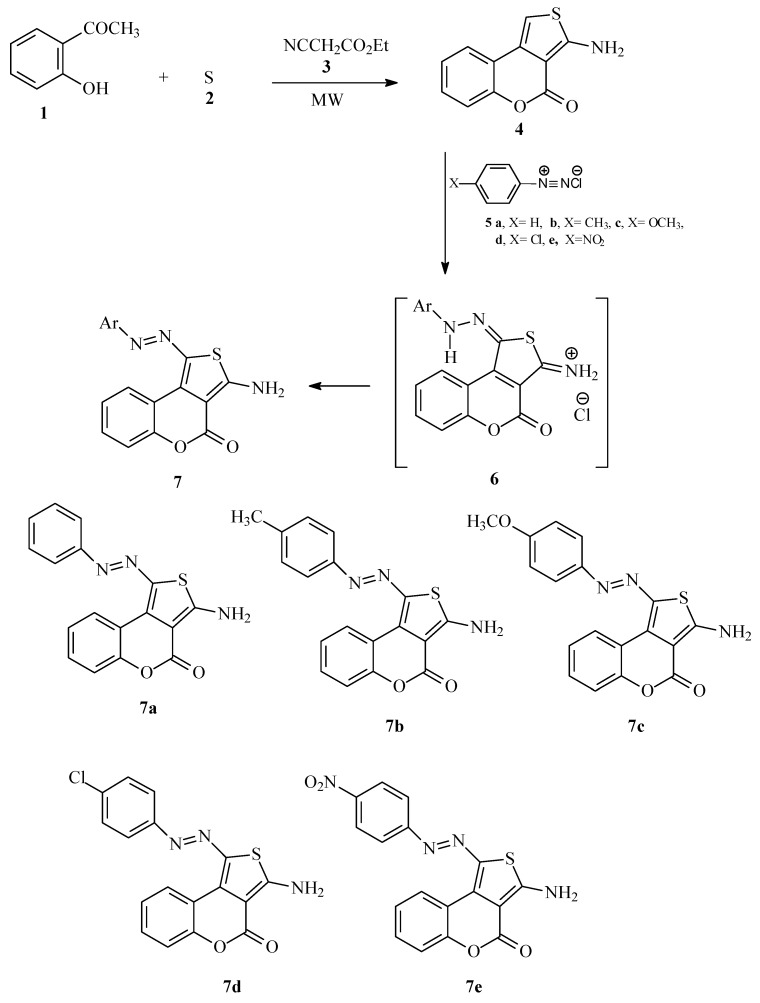
Preparation of monoazo disperse dyes **7a**–**e**.

Aminothienochromene **4** coupled with aryldiazonium chlorides to afford arylazothienochromene disperse dyes **7a**–**e**. These compounds are most likely formed via intermediacy of **6**. The structures of compounds **7a**–**e** (*cf*. Scheme 1) were established based on their IR spectra, which show absorption bands at *ca*. 3,370 and 3,270 cm^−1^ for amino groups, their ^1^H-NMR spectra, which displayed signals for aromatic protons along with a broad signal at *δ_H_* *ca*. 7 ppm (D_2_O exchangeable) for the two amino protons, and the absence of a thiophene 1-H singlet at *δ_H_* 6.86 ppm. 

When the disperse dyes **7a**–**e** were applied to polyester fabrics at 2% (dye shade), using the high temperature dyeing method (HT) at 130 °C, brown to violet color shades were obtained. The dyeing properties on the polyester fabrics were evaluated in terms of their fastness properties (e.g., fastnesses to washing, perspiration and light).

The surface color yield *K⁄S* was used to explain the amount of dye absorbed on the surface of the fabric. The *K⁄S* values listed in Table 1 show that arylazothienochromene disperse dyes showed the best build-up for polyester fabric and have high affinity for the polyester fabric, except for dyes **7b** and **7d** that showed poor affinity for the polyester fabric because of their lower molecular polarity.

The color of dyeing on polyester fabrics is expressed in terms of CIELAB values (Table 1), and the following CIELAB coordinates were measured: lightness (L*); chroma (C*); hue angle (*h*) from 0° to 360°, a*, whose value represents the degree of redness (positive) and greenness (negative); and b*, whose value represents the degree of yellowness (positive) and blueness (negative).

Hue *h* is one of the three basic characters for color. The *h* values in Table 1 show that almost all of the dyed polyester samples expressed the same hue except dye **7e** as violet color. In general, the color hues of the disperse dye **7e** on the polyester fabric shifted to the bluish directions; this was indicated by the negative value of b* = −11.47 (yellow–blue axis). 

The positive values of a* (red–green axis) indicated that the color hues of the disperse dyes **7a**–**d** on the polyester fabric shifted to the reddish directions.

**Table 1:** Shade and optical measurements of the azo disperse dyes on the polyester fabrics.

Dye No	Color on polyester (2% shade)	L*	a*	b*	C*	h*	*K/S*
**7a**	Orange	58.12	52.91	64.18	83.18	50.50	20.66
**7b**	Brown	59.74	36.18	42.69	55.96	49.71	8.55
**7c**	Dark orange	46.13	48.90	51.72	71.18	46.60	28.68
**7d**	Orange	59.26	52.96	49.46	72.46	43.04	11.86
**7e**	Violet	39.61	44.29	−11.47	45.75	345.48	8.87

The visible absorption data for the synthesized dyes were measured in DMF and are listed in Table 2. The absorption maxima of the synthesized dyes ranged from 491 nm to 662 nm. Within the series of azo disperse dyes investigated, the relation between the shift observed in the absorption maxima *λ_max_* and polar characteristics of substituent, may be summarized as follows: (a) with the introduction of OCH_3_, Cl groups in the *para*-position of the arylhydrazono moiety, a bathochromic shift could be observed, dye **7c**, and **7d** (*λ*_max_ 497 and 500 nm), were characterized small bathochromic shifts compared with dye **7a**, Δ*λ*_max_ = 7 and 10, respectively; (b) the incorporation of a NO_2_ group in dye **7e** gave a better exhaustion and noticeable depth of color, Δ*λ*_max_= 172; (c) the bathochromic shift accompanying the substituents in the diazo component was in the following order H → CH_3_ → OCH_3_ → Br → NO_2_.

The physical data for the dyed fabrics given in Table 2, shows that the disperse dyeing displayed moderate fastness levels to light. Although in all the cases light fastness was poor (Table 2), the dyes **7a** and **7e** were marginally superior to the others, emphasizing the importance of appropriate substitutents. Attempts are in hand to improve the light fastness properties of these dyes.

In addition the results obtained showed that dyed fabrics have excellent fastness levels to washing and perspiration fastness properties that may be due to: (a) the absence of solublizing groups, which renders solubility, and wash ability of the dye-out of the fabrics, (b) the size of the dye molecule is considered relatively big, (c) the good intra-fiber diffusion of the dye molecules inside the fabrics.

**Table 2:** Fastness properties of monoazo disperse dyes on polyester fabrics at 2% (o.w.f).

DyeNo	λ_max_(DMF)	Wash fastness			Perspiration fastness						Light fastness
					Alkaline			Acidic			
		Alt	SC	SW	Alt	SC	SW	Alt	SC	SW	
**7a**	490	5	5	5	5	5	5	5	5	5	3
**7b**	491	5	5	5	5	5	5	5	5	5	2
**7c**	497	5	4–5	4–5	5	4–5	5	5	4–5	5	2
**7d**	500	5	5	5	5	5	5	5	5	5	2–3
**7e**	662	5	5	5	5	5	5	5	5	5	3

Alt = alteration; SC = staining on cotton; SW = staining on wool.

## 3. Experimental

### 3.1. General

Melting points were recorded on a Gallenkamp apparatus. IR spectra were recorded using KBr pellets on a JASCO FTIR-6300 FT-IR spectrophotometer. ^1^H- and ^13^C-NMR spectra were recorded on Bruker DPX AvanceII 600 MHz super-conducting NMR spectrometer with proton spectra measured at 600 MHz and carbon spectra at 150 MHz, respectively. Mass spectra were measured on a high resolution GC/MS DFS-Thermo. Microanalyses were performed on Elementar-Vario Micro cube Analyzer. The dyeing of polyester fabrics were conducted using LINITEST + Laboratory High Temperature Dyeing and Fastness System (ATLAS, Lichtenstein, Germany). The colorimetric parameters of the dyed polyester fabrics were determined on a reflectance spectrophotometer (UltraScan PRO D65, HunterLab, Virginia, VA, USA). 

*3-Amino-4H-thieno[3,4-c]chromen-4-one* (**4**). A mixture of *o*-hydroxyacetophenone (**1**, 1.86 g, 10 mmol), sulfur (0.32 g, 10 mmol) and ethyl cyanoacetate (1.13 g, 10 mmol), in methanol (2 mL) was irradiated by focused microwaves at 50 °C for 4 h in the presence of diethylamine (0.73 g, 10 mmol). Completion of the reactions was monitored by TLC. The build-up of pressure in the closed reaction vessel was carefully monitored. After the irradiation, the reaction tube was cooled with high-pressure air through an inbuilt system in the instrument until the temperature had fallen below 50 °C. The mixtures were cooled and then poured into ice-water. The solids that formed were collected by using filtration and crystallized from benzene to give compound **4** as a yellow powder (48%), mp 199 °C (Lit. [16] mp 197–199 °C), IR (KBr): 3421, 3316 (NH_2_), 1684 (CO) cm^−1^. ^1^H-NMR (DMSO-d_6_): δ = 6.86 (1H, s, 1-H); 7.17–7.21 (m, 2H, 7-H and 9-H), 7.32 (t, 1H, *J* = 8.0 Hz, 8-H), 7.79 (brs. 2H, NH_2_, D_2_O-exchangeable), 7.83 (d, 1H, *J* = 6.8 Hz, 6-H). ^13^C-NMR (DMSO-d_6_): δ = 166.4 (C-3), 158.7 (C-2), 150.7, 130.7, 129.0, 124.1, 123.7, 117.8, 116.8, 97.8, 97.3. MS (EI) *m/z=* 217 (M]^+^, 100), Anal. Calcd for C_11_H_7_NO_2_S: C, 60.83; H, 3.23; N, 6.45; S, 14.75. Found: C, 60.88; H, 3.31; N, 6.45; S, 14.73. 

### 3.2. Reaction of Compound **4** with Aromatic Diazonium Salts

A solution of each aryldiazonium chloride (10 mmol, prepared as described earlier [14]) at 0 °C was added to a solution of **4** (10 mmol) in acetic acid (50 mL) containing sodium acetate (0.60 g). The reaction mixture was stirred at room temperature for 1 h and the solid product was collected by filtration and crystallized from DMF/ethanol (3:1).

*3-Amino-1-(phenyldiazenyl)-4H-thieno[3,4-c]chromen-4-one* (**7a**). Red crystals (79%), mp 239 °C (Lit. [5] mp 238–239 °C), IR (KBr): 3310 and 3164 (NH_2_), 1674 (CO) cm^−1^. ^1^H-NMR (DMSO-*d*_6_): δ = 7.02 (br s, 2H, NH_2_), 7.36–7.40 (m, 2H, arom-H), 7.44 (t, 1H, *J* = 8.4 Hz, arom-H), 7.50 (t, 2H, *J* = 7.8 Hz, arom-H), 7.58 (t, 1H, *J* = 7.2 Hz, arom-H), 7.74 (d, 2H, *J* = 8.4 Hz, arom-H), 8.83 (d, 1H, *J* = 9.0 Hz, arom-H). ^13^C-NMR (DMSO-d_6_): δ = 169.7 (CO), 158.7, 153.1, 152.5, 135.4, 133.5, 132.0, 129.9, 129.5, 129.3, 125.6, 122.2, 117.7, 117.5, 100.8. MS (EI) *m/z=* 321 (M]^+^, 100), UV/Vis at *λ*_max_(DMF) = 490 nm. Anal. Calcd for C_17_H_11_N_3_O_2_S: C, 63.53; H, 3.45; N, 13.07; S, 9.97. Found: C, 63.22; H, 3.51; N, 12.86; S, 10.04.

*3-Amino-1-(p-tolyldiazenyl)-4H-thieno[3,4-c]chromen-4-one* (**7b**). Brown powder (75%), mp 293 °C (Lit. [14] mp 290–292 °C), IR (KBr): 3386 and 3261 (NH_2_), 1683 (CO) cm^−1^; ^1^H-NMR (DMSO-*d*_6_): δ = 2.37 (s, 3H, CH_3_), 6.96 (brs, 2H, NH_2_), 7.34–7.35 (m, 3H, arom-H), 7.41 (t, 1H, *J* = 8.4 Hz, arom-H), 7.55 (t, 1H, *J* = 7.2 Hz, arom-H), 7.62 (d, 2H, *J* = 8.4 Hz, *p*-tolyl-H), 8.80 (d, 1H, *J* = 7.8 Hz, arom-H). ^13^C-NMR (DMSO-*d*_6_): δ = 168.4 (CO), 158.7, 153.0, 150.5, 139.5, 134.5, 133.6, 131.8, 130.4, 129.2, 125.5, 122.2, 117.7, 117.6, 100.5, 21.4 (CH_3_). MS (EI) *m/z=* 335 (M]^+^, 100), UV/Vis at *λ*_max_(DMF) = 319 nm. Anal. Calcd for C_18_H_13_N_3_O_2_S: C, 64.46; H, 3.90; N, 12.52; S, 9.56. Found: C, 64.76; H, 3.99; N, 12.63; S, 9.32. 

*3-Amino-1-((4-methoxyphenyl)diazenyl)-4H-thieno[3,4-c]chromen-4-one* (**7c**). Reddish brown crystals (68%), mp 281–283 °C (Lit. [14] mp 280–281 °C), IR (KBr): 3380 and 3260 (NH_2_), 1681 (CO) cm^−1^. ^1^H-NMR (DMSO-*d_6_*): δ = 3.84 (s, 3H, OCH_3_), 6.96 (br s, 2H, NH_2_), 7.07 (d, 2H, *J* = 9 Hz, *p*-methoxyphenyl-H), 7.32 (d, 1H, *J* = 8.4 Hz, arom-H), 7.39 (t, 1H, *J* = 7.8 Hz, arom-H), 7.53 (t, 1H, *J* = 8.4 Hz, arom-H), 7.70 (d, 2H, *J* = 9 Hz, *p*-methoxyphenyl-H), 8.79 (d, 1H, *J* = 8.4 Hz, arom-H). ^13^C- NMR (DMSO-*d*_6_): δ = 168.0 (CO), 160.7, 158.8, 152.9, 146.6, 133.8, 133.3, 131.6, 129.1, 125.5, 123.9, 1117.7, 117.6, 115.2, 100.1, 55.9 (OCH_3_). MS (EI) *m/z=* 351 (M]^+^, 100), UV/Vis at *λ*_max_(DMF) = 497 nm. Anal. Calcd for C_18_H_13_N_3_O_3_S: C, 61.52; H, 3.72; N, 11.95; S, 9.12. Found: 61.25; H, 3.77; N, 11.88; S, 8.79.

*3-Amino-1-((4-chlorophenyl)diazenyl)-4H-thieno[3,4-c]chromen-4-one* (**7d**). Red crystals (77%), mp 304 °C (Lit. [14] mp 300–302 °C), IR (KBr): 3377 and 3270 (NH_2_), 1689 (CO) cm^−1^. ^1^H-NMR (DMSO-*d_6_*): δ = 7.34 (d, 1H, *J* = 8.4 Hz, arom-H), 7.40 (t, 1H, *J* = 7.8 Hz, arom-H), 7.54 (d, 2H, *J* = 9.0 Hz, arom-H), 7.57 (t, 1H, *J* = 7.8 Hz, arom-H), 7.70 (d, 2H, *J* = 9.0 Hz, arom-H), 8.78 (d, 1H, *J* = 8.7 Hz, arom-H), 9.51 (br s, 2H, NH_2_). ^13^C-NMR (DMSO-*d*_6_): δ = 169.0 (CO), 158.6, 153.2, 151.2, 136.2, 133.4, 133.3, 132.2, 129.9, 129.3, 125.6, 123.6, 117.7, 117.4, 101.2. MS (EI) *m/z =* 355 (M]^+^, 100), UV/Vis at *λ_max_*(DMF) = 500 nm. Anal. Calcd for C_17_H_10_ClN_3_O_2_S: C, 57.38; H, 2.83; N, 11.81; S, 9.01.Found: C, 57.48; H, 3.02; N, 11.97; S, 8.77.

*3-Amino-1-((4-nitrophenyl)diazenyl)-4H-thieno[3,4-c]chromen-4-one* (**7e**). Violet crystals (75%), mp 307 °C (Lit. [14] mp 302–304 °C), IR (KBr): 3373 and 3264 (NH_2_), 1689 (CO) cm^−1^. ^1^H-NMR (DMSO-*d_6_*): δ = 7.42 (d, 1H, *J* = 7.2 Hz, arom-H), 7.46 (t, 1H, *J* = 7.6 Hz, arom-H), 7.65 (t, 1H, *J* = 8.8 Hz, arom-H). 7.86 (d, 2H, *J* = 7.2 Hz, 4-nitrophenyl-H), 7.95 (br s, 2H, NH_2_), 8.32 (d, 2H, *J* = 8.8 Hz, 4-nitrophenyl-H), 8.86 (d, 1H, *J* = 8 Hz, arom-H). MS (EI) *m/z=* 366 (M]^+^, 100), UV/Vis at *λ_max_*(DMF) = 662 nm. Anal. Calcd for C_17_H_10_N_4_O_4_S: C, 55.73; H, 2.75; N, 15.29; S, 8.75. Found: 55.87; H, 2.91; N, 15.29; S, 8.41.

### 3.3. High Temperature Dyeing Method (HT)

#### 3.3.1. Materials

Polyester 100% (150 130 g/m^2^, 70/2 denier) was obtained from El-Shourbagy Co., Cairo, Egypt. The fabric was treated before dyeing with a solution containing non-ionic detergent (Sera Wash M-RK, 5 g/L) and sodium carbonate (2 g/L) in a ratio of 50:1 at 60 °C for 30 min, then thoroughly washed with water and air dried at room temperature.

#### 3.3.2. Dyeing

The dye bath (1:50, good to dye liquor ratio) is a sealed stainless steel dye pot of 300 mL capacity in the “Atlas-Germany” dyeing machine. An additional dispersing agent (Sodium lignin sulfonate) was added with a ratio to dye 1:1 and the pH of the bath was adjusted to 4.5–5 using glacial acetic acid. The dyeing was being carried out by raising the dye bath temperature from 20 °C to 130 °C at a rate of 7 °C/min and holding at this temperature for 60 min before rapidly cooled to 50 °C. The dyed fabrics was then rinsed with cold water, reduction-cleared using sodium hydroxide (2 g/L) and sodium hydrosulphite (2 g/L) at 80°C for 30 min and then the dyed samples thoroughly washed and air-dried.

### 3.4. Color Measurements and Analyses

#### 3.4.1. Color Measurements

The colorimetric parameters (Table 1) of the dyed polyester fabrics were determined on a reflectance spectrophotometer. The color yields of the dyed samples were determined by using the light reflectance technique performed on UltraScan PRO D65 UV/VIS Spectrophotometer. The color strengths, expressed as K/S values, were determined by applying the Kubelka-Mink equation as follows:

K/S = [(1 − R) ^2^/2R] − [(1 − R_o_) ^2^/2R_o_]

where *R* = decimal fraction of the reflectance of the dyed fabric; *R_o_* = decimal fraction of the reflectance of the undyed fabric; *K* = absorption coefficient; *S* = scattering coefficient.

#### 3.4.2. Fastness Testing

Fastnesses to washing, perspiration, and light were tested according to reported methods [17].

## 4. Conclusions

In summary, a series of monoazo disperse dyes were synthesized from 3-aminothienochromene. Synthesis of the aminothienochromene in a microwave oven afforded better yield and shorter reaction times. The dyes produced in this manner were then applied to polyester fabrics using the high temperature dyeing method at 130 °C. The dyed polyester fabrics, which displayed brown to violet hues, showed excellent washing and perspiration fastness and moderate light fastness. 
